# Regarding: “Exercise Stress Echocardiography Predicts Adverse Cardiovascular Events in Hypertrophic Cardiomyopathy: A 5-Year Prospective Study”

**DOI:** 10.31083/RCM49547

**Published:** 2026-03-09

**Authors:** Ye Su, Qionghui Peng, Lixue Yin, Chunmei Li

**Affiliations:** ^1^Department of Cardiovascular Ultrasound & Noninvasive Cardiology, Sichuan Provincial People's Hospital, University of Electronic Science and Technology of China, 610031 Chengdu, Sichuan, China; ^2^Ultrasound Medicine and Computational Cardiology Key Laboratory of Sichuan Province, Sichuan Provincial People's Hospital, University of Electronic Science and Technology of China, 610031 Chengdu, Sichuan, China

Dear Editor,

We sincerely thank the authors of the Letter to the Editor for their thoughtful engagement with our recent publication and for their constructive suggestions, which aim to enhance the methodological transparency and clinical applicability of our work. We appreciate their recognition of our findings and welcome this opportunity to clarify key aspects of our study design.

In response to the specific points raised:

(1) Equipment, software, and Frame rate.

We fully agree with the importance of standardized imaging protocols in longitudinal strain studies. In this study, the hypertrophic cardiomyopathy (HCM) patients and the control group were positioned in the left lateral position, and their electrocardiogram was synchronously recorded. For rest and peak stress Echocardiography, we used the GE-Vivid E95 color Doppler ultrasonic diagnostic instrument (E95, GE Medical Systems, Milwaukee, WI, USA), and the 4V-D full volume probe (frequency 1.5–4.0 MHz, GE Medical Systems, Milwaukee, WI, USA), Philips-EPIQ7C ultrasonic diagnostic instrument (EPIQ7C, Philips Healthcare, Netherlands), and the X5-1 fully functional pure wave single crystal matrix probe (frequency 1.0–5.0 MHz, Philips Healthcare, Netherlands). Apical four-chamber, three-chamber, two-chamber, and apical full-volume dynamic images of at least five cycles at rest were collected. Apical four-chamber, three-chamber, and two-chamber dynamic images of at least five cycles at the peak stage were collected. Dynamic images were acquired at a fixed frame rate ≥60 frames per second.

Image Data Analysis: QLAB quantitative analysis software (13.0, Philips Healthcare, Netherlands) and ECHOPAC analysis software (203, GE Medical Systems, Milwaukee, WI, USA) were used. All measurements adhered to the American Society of Echocardiography (ASE) guidelines [1], and left atrial strain (LAS) analysis followed the consensus standardization established by the EACVI/ASE/Industry Task Force [2]. Throughout the five-year follow-up period, the same ultrasound equipment and software versions were consistently used to minimize technical variability.

(2) Regarding the exploration of a clinically interpretable LAS cut-off value and comparison with the 2014 European Society of Cardiology (ESC) HCM-Risk sudden cardiac death (SCD) model [3].

We appreciate the suggestion to identify a specific LAS cut-off value to facilitate clinical translation. Receiver operating characteristic (ROC) analysis in our cohort identified R_4_D_LASr ≤14.50% as an optimal cut-off, demonstrating a sensitivity of 90.00% and specificity of 85.29% (area under the curve, AUC = 0.93) for predicting 5-year adverse cardiovascular events. Similarly, P_LASr_ED ≤16.84% also exhibited strong predictive performance (AUC = 0.89).

Regarding comparison with the 2014 ESC HCM Risk-SCD model: the 2014 ESC risk model is predominantly built upon static, structural risk markers, including a family history of SCD and the presence of non-sustained ventricular tachycardia. In our cohort, due to multiple factors including diverse social and family backgrounds, economic status and personal awareness, implementation of this model was constrained by significant gaps in patient-reported clinical data: approximately 87% of participants could not specify their disease onset date, 76% lacked reliable information on a family history of SCD or syncope, and 81% were unsure about previous episodes of non-sustained ventricular tachycardia. Therefore, a comparative analysis between our proposed model and the 2014 ESC HCM Risk-SCD Model [3] cannot be performed at this stage. In real-world clinical practice, a significant proportion of patients with HCM are unable to provide complete personal medical history or detailed family history information, which often renders traditional risk models inapplicable. In contrast, exercise stress echocardiography-left atrial strain offers dynamic, functional insights as an objective imaging biomarker that does not rely on patients’ subjective recall or complex familial investigations.

The study is still ongoing. In future studies, we will expand the sample size, enrich the genetic subtypes and classification of HCM, incorporate more sensitive and easily measurable parameters, use a more complete clinical data set, and conduct continuous validation. We hope that this method can become a feasible supplementary strategy for HCM risk stratification and can identify high-risk patients earlier.

(3) Investigating whether the change rate of LAS from rest to post-exercise provides superior prognostic information.

We thank the reviewers for suggesting the potential utility of the LAS change from rest to peak exercise as a marker of atrial reserve. This is an excellent direction for future research, and we are planning a follow-up study to explore dynamic strain parameters in HCM.

(4) For real-world patients who cannot provide a complete medical history, could you propose a simplified approach to integrate LAS with the ESC model (e.g., prioritizing the most critical ESC risk factors when incorporating LAS)?

We intend to adopt the following strategy: combined assessment of core ESC factors plus LAS. For HCM patients with incomplete medical histories, the algorithm is as follows: first, collect information on 1–2 core ESC risk factors whenever feasible. Second, perform exercise stress echocardiography and measure resting three-dimensional left atrial reservoir strain (R_4_D_LASr). Third, integrate these findings with the identified ESC risk factors. If a patient presents with any one of the core ESC risk factors accompanied by a significant reduction in R_4_D_LASr (e.g., ≤14.50%), this combination may serve as an enhanced risk stratification signal. The application of this “ESC factors plus novel functional parameter” model can facilitate clinicians in implementing more proactive management strategies, such as shortening follow-up intervals (e.g., from the conventional 12 months to 6 months), and recommending more comprehensive examinations (e.g., cardiac magnetic resonance imaging, genetic testing). In clinical practice with limited available information, leveraging the most accessible and critical ESC risk markers supplemented by the LAS parameter enables rapid and effective preliminary risk screening, thereby guiding subsequent precise assessments without missing high-risk patients.

(5) What simple, standard ways will you use in future studies to collect this missing information (e.g., structured forms or checking electronic health records)?

We will adopt structured electronic data collection forms embedded within the electronic health record (EHR) system, ensuring that key information (e.g., family history, symptom history, medication history) is systematically documented at the initial patient visit. Concurrently, we will establish a regular data verification mechanism to minimize the loss of information through system-generated reminders and manual audits. Additionally, patient self-administered questionnaires (available in both electronic and paper formats) will be designed for completion during waiting periods and subsequently reviewed by medical staff for accuracy.

(6) Did all doctors and analysts get the same training to do echocardiograms and measure LAS? What did this training involve (e.g., practicing with a reference team)?

Yes, all investigators received standardized training. Our department serves as the Sichuan Provincial Quality Control Center for Ultrasound Medicine and the Sichuan Key Laboratory of Echocardiography, Electrophysiology, and Biomechanics. The training encompassed: operational protocols for ultrasound equipment, standardized procedures for image acquisition, LAS measurement methodology in accordance with the guidelines from the EACVI/ASE/Industry Task Force [2], and hands-on training sessions with regular quality control meetings.

(7) How did you make sure images were clear during peak exercise, when patients tend to move more? Did you exclude blurry images, and if so, how many?

Prior to exercise, all participants received personalized training on breathing control and breath-holding techniques along with preliminary image acquisition. During exercise, image capture was synchronized with short-duration breath-holding to minimize interference from lung aeration, which significantly improved the quality and success rate of image acquisition. All images were real-time assessed for quality by senior cardiologists. If an image was blurred or non-trackable, immediate re-acquisition was performed. A total of 8 patients were excluded from the study due to suboptimal image quality at the screening stage; these patients did not enter the final analysis cohort.

(8) Your LAS cut-offs (R_4_D_LASr ≤14.50% and P_LASr_ED ≤16.84%) work well for predicting events, but do these cut-offs work equally well for different types of HCM patients (e.g., those with/without blocked heart flow, different age groups, or patients with other illnesses like high blood pressure)?

In the current study, subgroup analyses were conducted for obstructive and non-obstructive HCM. The results showed that LAS was significantly reduced in both subgroups and correlated with adverse clinical events. The proposed LAS cutoff values exhibited predictive validity in both obstructive and non-obstructive HCM patients. The study is currently ongoing. We are also enrolling HCM patients with comorbidities and across different age groups, aiming to perform more detailed and in-depth analyses, which will further refine our findings in subsequent research.

(9) How should doctors handle patients with LAS values close to the cut-off (e.g., 14.0–15.0%)? Should they repeat the test or do extra checks (e.g., genetic testing)?

For patients with LAS values in the borderline range, repeated measurements and dynamic monitoring are recommended. If the repeat results remain borderline, this can be regarded as a “gray zone”. It is advisable to intensify clinical surveillance within 3–6 months (e.g., performing exercise stress echocardiography, ambulatory electrocardiography, and conducting close follow-ups) to monitor the changing trend of LAS. Additionally, multimodal assessment integration is recommended, which includes detailed collection of symptoms and family history combined with ambulatory electrocardiography and cardiac magnetic resonance imaging to improve the overall efficacy of risk stratification. If the patient presents with high-risk factors such as syncope, ventricular tachycardia, family history of SCD, or significant abnormalities on cardiac magnetic resonance imaging, LAS may be considered as an enhanced risk stratification signal, and genetic testing can be performed to further confirm the diagnosis and assess genetic risk [4, 5, 6]. Genetic testing should not be conducted solely based on borderline LAS values.

Once again, we express our gratitude for the constructive feedback, which has helped refine our work and outline meaningful directions for future investigation. We are committed to further validating these findings in larger and more diverse cohorts and integrating multimodal imaging into the comprehensive risk stratification framework of HCM.
